# [Retracted] Curcumin improves the paclitaxel-induced apoptosis of HPV-positive human cervical cancer cells via the NF-κB-p53-caspase-3 pathway

**DOI:** 10.3892/etm.2025.12902

**Published:** 2025-06-10

**Authors:** Yu-Ping Dang, Xiao-Ying Yuan, Rong Tian, Dong-Guang Li, Wei Liu

Exp Ther Med 9:1470–1476, 2015; DOI: 10.3892/etm.2015.2240

Following the publication of the above paper, it was drawn to the Editor’s attention by a concerned reader that, regarding the flow cytometric plots shown in Fig. 1A on p. 1472, the data panels appeared to show similar groupings of dots, which would not have been anticipated if these experiments had been performed discretely under different experimental conditions, suggesting a fundamental flaw either in the way in which these experiments were performed, or in how the results were outputted.

The Editor of *Experimental and Therapeutic Medicine* has decided that this paper should be retracted from the Journal on account of a lack of confidence in the presented data. The authors were asked for an explanation to account for these concerns, but the Editorial Office did not receive a reply. The Editor apologizes to the readership for any inconvenience caused.

